# Thermally Treated Waste Silt as Filler in Geopolymer Cement

**DOI:** 10.3390/ma14175102

**Published:** 2021-09-06

**Authors:** Abbas Solouki, Alireza Fathollahi, Giovanni Viscomi, Piergiorgio Tataranni, Giovanni Valdrè, Stephen J. Coupe, Cesare Sangiorgi

**Affiliations:** 1 Department of Civil, Chemical, Environmental and Materials Engineering, University of Bologna, Via Terracini 28, 40131 Bologna, Italy; piergiorg.tataranni2@unibo.it (P.T.); Cesare.sangiorgi4@unibo.it (C.S.); 2 Centre for Agroecology Water and Resilience (CAWR), Coventry University, Wolston Lane, Ryton-on-Dunsmore CV8 3LG, UK; ad2068@coventry.ac.uk (A.F.); steve.coupe@coventry.ac.uk (S.J.C.); 3 Bologna Strade, Via Zanardi 372/2, 40131 Bologna, Italy; viscomi@bolognastrade.it; 4 Department of Biological, Geological, and Environmental Sciences, University of Bologna, Piazza di Porta San Donato 1, 40126 Bologna, Italy; giovanni.valdre@unibo.it

**Keywords:** quarry waste, silt calcination, ambient temperature curing, aggregate recycling, DOE, DSLT

## Abstract

This study aims to investigate the feasibility of including silt, a by-product of limestone aggregate production, as a filler in geopolymer cement. Two separate phases were planned: The first phase aimed to determine the optimum calcination conditions of the waste silt obtained from Società Azionaria Prodotti Asfaltico Bituminosi Affini (S.A.P.A.B.A. s.r.l.). A Design of Experiment (DOE) was produced, and raw silt was calcined accordingly. Geopolymer cement mixtures were made with sodium or potassium alkali solutions and were tested for compressive strength and leaching. Higher calcination temperatures showed better compressive strength, regardless of liquid type. By considering the compressive strength, leaching, and X-ray diffraction (XRD) analysis, the optimum calcination temperature and time was selected as 750 °C for 2 h. The second phase focused on determining the optimum amount of silt (%) that could be used in a geopolymer cement mixture. The results suggested that the addition of about 55% of silt (total solid weight) as filler can improve the compressive strength of geopolymers made with Na or K liquid activators. Based on the leaching test, the cumulative concentrations of the released trace elements from the geopolymer specimens into the leachant were lower than the thresholds for European standards.

## 1. Introduction

Nowadays, sustainability is a basic principle that is considered by politicians and many organizations in society. As for the construction sector, there is a growing interest in manufacturing sustainable buildings and infrastructure with high percentages of recycled materials. However, gravel quarries are still operative, and natural aggregate production requires landfill management of the waste/by-products. Annually, the construction sector demands about 3000 million tons of non-renewable natural aggregates. In 2018, for instance, mining and quarrying waste exceeded 623 million tons [1,2].

During the aggregate manufacturing process, water is used to wash the surface of the aggregates clean of dirt and mud. The water is then pumped out to sedimentation lakes or mine tailings nearby. For instance, during limestone production silt and clay particles are the main substances found in sedimentation lakes. Clay minerals have the smallest particle size compared to silt and sand. In general, clay particles are formed from two main crystal layers of silica (tetrahedral) and alumina (octahedral), and their configuration, bonding type, and metallic ions in the crystal lattice characterizes and separates different clay particles from one another [3]. The materials stored and kept in the sedimentation lakes could become an environmental issue.

Different methods and approaches have been developed over the past few years to reduce the undesirable impact of quarry waste on the environment. For instance, quarry waste has been used in cement mortars [4,5,6] and asphalt pavements [7,8,9]. A different approach includes recycling mineral fillers and quarry dust in geopolymer cement production [10,11,12,13,14].

Geopolymer cement is an alternative binder to ordinary Portland cement, which was first introduced by Davidovits in the 1970s during his efforts to produce nonflammable and noncombustible plastics. Geopolymer cements are materials that are rich in aluminum silicates, which are transformed into a tridimensional tecto-aluminosilicate structure in an alkaline solution. Their geosynthesis is based on the ability of the aluminum ion (6-fold or 4-fold coordination) to induce crystallographical and chemical changes in the silica backbone [15,16]. A geopolymer cement could be made by adding alkali solutions to materials rich in aluminosilicates (such as metakaolin and fly ash).

Various studies have investigated the possibility of using clay and silt substances for geopolymer cement production. For instance, clay and fly ash were used as precursors to produce sustainable geopolymer bricks [17]. For this purpose, 11 different mixtures were produced by substituting different percentages of fly ash (0–100%) with clay. The results indicated that bricks created with 30–60% fly ash had promising physical properties and mechanical strength. In a different approach, Lampris et al. collected waste silt from different washing plants in the UK, which was mixed with metakaolin and fly ash to produce geopolymers [18]. The room temperature curing of the sample made entirely of silt reached 18.75 MPa after 7 days, whereas samples made with silt and metakaolin showed a higher compressive strength of 30.5 Mpa. The addition of metakaolin and fly ash improved the geopolymer reaction/process, which led to higher compressive strength. The authors suggested that the strength of silt-based geopolymers was enough to be used as aggregates in unbound applications. 

During geopolymer cement production, small grain-sized aggregates, which have a certain quantity of reactivity, are added to reduce brittleness and to minimize the pore size and shrinkage values of the final mixture. These materials, which are referred to as fillers, partially react with the geopolymer matrix, producing stronger networks [15]. For instance, limestone, marble, and basalt waste powders were used to produce geopolymer composites [19]. The described study claimed that the usage of up to 50% limestone or marble waste powder increased the sample strength. Overall, all of the mentioned waste powders positively affected the overall strength, abrasion, and water absorption of the geopolymer samples. Various inert and/or partially reactive waste materials have been used as fillers. The effect of different fillers such as quartz fume, illitic clay, and recycled chamotte material on the thermo-mechanical properties of geopolymer was studied [20]. The highest compressive strength for the samples made with clay was obtained when 10% clay was used as a filler. However, the best performance in terms of lower porosity and higher strength was observed when 20% quartz or chamotte was used in the mixture. In a similar study, calcined kaolinitic claystone and potassium silicate hardeners were used to produce geopolymer samples [21]. Quartz, corundum, chamotte, and cordierite were used as fillers. The authors claimed that the viscosity of such mixes was low enough to allow the incorporation of up to 65% of filler. The geopolymer samples had a stable structure at an elevated temperature of 1000 °C, and the shrinkage of the geopolymer samples significantly reduced at high temperatures when the filler was used. The best fillers were corundum or chamotte, which performed better compared to the other types of fillers [21]. 

In some studies, the silt and clay materials underwent thermal pretreatments. Due to the mineralogy and nature of clay, pretreatments could increase the reactivity of the precursors by altering their mineralogical properties. For example, a case study was conducted in a reservoir located in southern Italy that aimed to tackle the loss of water storage capacity in the lakes where the alkaline activation of silt residues was suggested as a solution. The results indicated that the clay and silt could be calcined and reused in applications such as a binder, precast elements, and bricks through alkaline activation [22]. The calcination of quarry dust reduces the crystalline structure and improves the reactivity of mineral fillers. Calcination requires the quarry dust to be heated to 600–800 °C for an average duration of 2 h [23]. However, the calcination temperatures could vary based on the mineralogy, type, or physical properties of quarry waste. For instance, the surface area and particle size were used as parameters to determine the optimum calcination temperature of low-grade clay [24]. The largest specific surface area of 18.43 m^2^/g and the smallest median particle size of 16.4 µm were achieved at 550 °C. Therefore, the calcination temperature of 550 °C was selected as the most efficient temperature for the thermal treatment of the low-grade clay.

Quarry waste management has become an important aspect over the past few decades, and various methods have been proposed to reduce its impact on the environment. In this regard, geopolymer cement has become an interesting and alternative method for aggregate by-product management. In most cases, waste minerals have been used as precursors in geopolymer production, while the possibility of using the by-products as filler has received less attention. Depending on the mineralogy of the mineral fillers, some may be less reactive and a have low Si and/or Al content, which may not be suitable for use as precursors. However, these materials may have promising performance if used as fillers in geopolymer mixtures. Thus, the main aim of the current study was to investigate the feasibility of including silt (a by-product of limestone aggregate production) in geopolymer cement production as a filler.

Furthermore, considering their final possible application as construction materials, leaching tests were performed. Construction and/or pavement products are indeed constantly in contact with stormwater produced by rainfall events. These products experience varying stormwater conditions during their lifespan, including acidic or basic pH and different temperatures, depending on the time of the year [25]. This exposure may lead to the release of various organic and inorganic compounds into the stormwater, including heavy metals, nutrients, and, for example, polycyclic aromatic hydrocarbons (PAHs) [26,27]. Previous studies have shown that the scale of contaminants released into stormwater from construction materials is comparable to pesticide contamination in agricultural systems [25,28]. Therefore, the potential of new construction and paving products to release organic and inorganic compounds into water bodies should be evaluated before their application in roads and buildings. Thus, in this study, the potential of a geopolymer to release harmful compounds was investigated through leaching tests according to the European Commission (EC) Construction Products Regulation (CPR).

## 2. Materials and Methods

Two separate phases were planned for the current study. For the first phase, the aim was to determine the optimum calcination conditions of the waste silt obtained from S.A.P.A.B.A. s.r.l. (Società Azionaria Prodotti Asfaltico Bituminosi Affini) sedimentation lakes in Italy. The second phase focused on determining the optimum amount of silt (%) that could be used in geopolymer cement mixture.

For the first phase, a DOE (design of experiment) was produced, and raw silt was calcined accordingly. A DOE is a branch of applied statistics that is used to plan, conduct, and analyze the effect of different input variables on the desired outcome(s) of a test or process. The selected input variables for the first phase were the calcination temperature and the calcination time, where the outcome was selected as the unconfined compressive strength (UCS). The DOE analysis indicated the best calcination time and temperature that produced the highest strength amongst all of the samples. The obtained data were then compared with mineralogical and environmental tests to determine the optimum calcination conditions of the waste silt.

**Figure 1:**
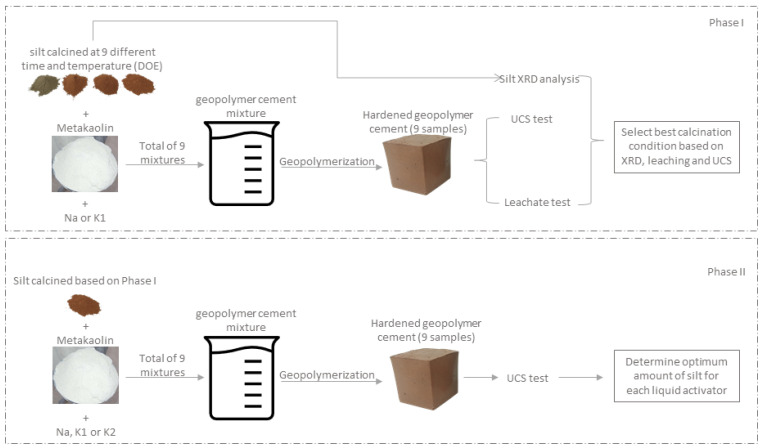
Methodology flow chart.

The second phase, independent of the first section, focused on determining the optimum amount of silt that could be used in geopolymer cement mixtures. However, the optimum silt calcination conditions were taken from the first phase. Consequently, an additional DOE was designed, where the silt and activator type were selected as variables, each having 3 levels/types. The UCS was selected as the outcome variable. Thus, the effect of the activator type and the silt percentage on the final UCS was studied. The overall workflow for the methodology is depicted in Figure 1.

### 2.1. Silt Characterization and Thermal Treatment (Phase I)

**Figure 2:**
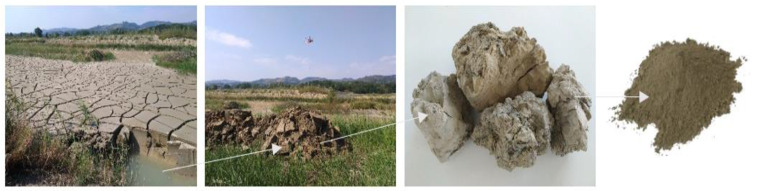
The washed-off silt during limestone production is stored at S.A.P.A.B.A. s.r.l.’s sedimentation lakes, where it is collected and processed for further use [6].

**Figure 3:**
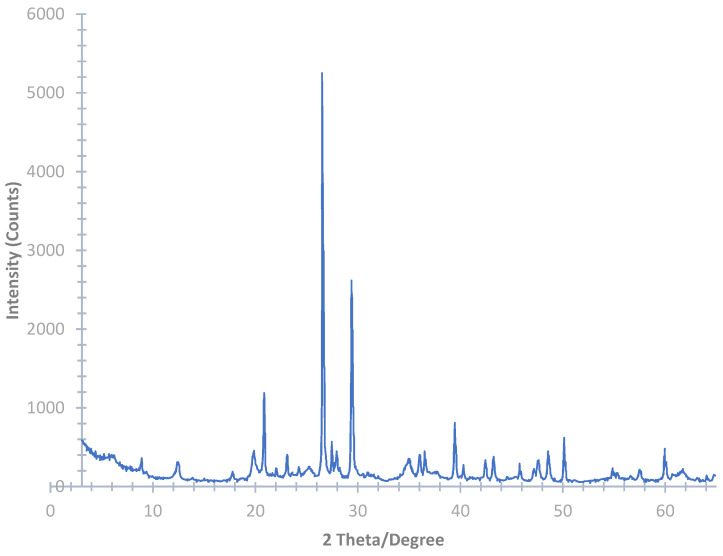
XRD of the uncalcined silt.

During aggregate production, undesirable substances such as silt, clay, and dirt are washed and separated during the washing process. The substances are then pumped out of the plant and stored in a sedimentation lake. For the current study, the silt was excavated from S.A.P.A.B.A. s.r.l.’s sedimentation lakes (Bologna, Italy). The silt was then oven-dried, sieved, and crushed to a fine powdery state using the Los Angles machine (Figure 2). The materials used in geopolymer cement/concrete should be rich in both aluminum and silicates. The aluminosilicates can react with the liquid hardeners and can produce a geopolymer binder. Thus, the precursors and fillers should have both an appropriate mineralogy and chemical composition. Thus, the chemical composition of the silt was determined by an Ecamricert X-ray fluorescence spectrometer (Shimadzu, Kyoto, Japan) (Table 1), and the mineralogical analysis (Figure 3) was conducted using a Philips diffractometer (Cu Kα radiation, graphite monochromator on the diffracted beam, power supply of 40 kV and 30 mA, step size of 0.02° 2θ, integration time of 2.3 s/step, range 3°–65° 2θ) [29].

Semi-quantitative evaluation of the mineralogical phases was performed using the XPowder computer program (Rigaku, Tokyo, Japan) and the reference intensity ratios (RIR) method (Chung 1974) with a quasi-random specimen preparation [30].

The initial data indicated a presence of 43.5% and 12.5% SiO_2_ and Al_2_O_3_, respectively (Table 1). However, the mineralogical data indicated that the silt had a high crystallinity, which was composed of minerals including quartz, calcite, phyllosilicates with characteristic interplanar distances associable with chlorite, kaolinite/serpentine, illite/mica, feldspar such as albite and K-feldspar, and traces of dolomite (Figure 3).

**Figure 4:**
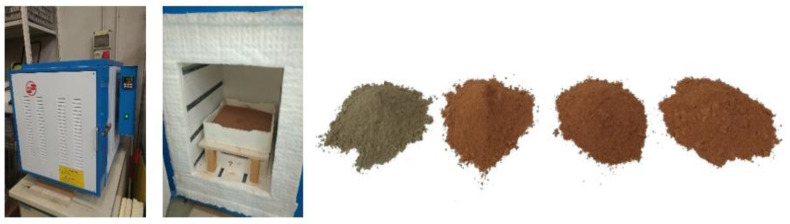
Silt calcination process. Different calcination temperatures change the physical appearance of the silt. From left to right, the images show the uncalcined silt and the silt calcined at 550, 750, and 850 °C.

To increase the reactivity of the silt, thermal treatment (calcination) was conducted using a static furnace (Pixsys ATR621, Venice, Italy) with a heating rate of 10 °C/min. To proceed with the calcination, a design of experiments (DOE) was established using JMP^®^ software (Version 14.0. SAS Institute Inc., Cary, NC, USA, 1989–2019). The response surface method has gained popularity over time and has turned into a common mathematical and statistical tool for optimizing processes and evaluating the relationships between various input factors and responses. Moreover, this method produces reliable results and can decrease the number of tests required, reducing the needed time and expenses. Thus, a response surface method (RSM) was applied, where time and temperature were selected as the two independent factors, and the response was the unconfined compressive strength (UCS). A stepwise approach the using minimum BIC (direction forward, no rules) was used to build the final second-degree model. Consequently, a total of nine runs were produced, each with different times and temperatures for the calcination process (Table 2). The temperatures ranged between 200 and 850 °C, and the time was selected to be between 1 to 12 h. The runs were randomized to reduce type II errors as much as possible. The calcined silt obtained from each run was used to produce geopolymer cement samples. Each produced mixture was then tested for UCS. The calcination process is shown in Figure 4.

### 2.2. Geopolymer Binder Preparation and Testing Procedures (Phase I)

#### 2.2.1. Sample Preparation

Sodium-based and commercially available potassium-based liquid hardeners were used to produce the geopolymer cement mixtures. A sodium-based solution (labeled “Na”) was prepared by mixing five parts sodium silicate (MR = 1.99) with one part NaOH solution (10 molars) (NaOH 98% purity, thermofisher). The potassium-based solution (labeled “K1”) was a commercial product with an MR of 1.7. As for the main precursor, a highly reactive metakaolin (labeled “MK”) was used. Regardless of the type of liquid hardener, cubic samples were prepared by mechanically mixing metakaolin with the liquid solution (sodium or potassium) for 10 min. The calcined silt was added, and the mixing continued for an additional 5 min. The mixture was then poured into cubic Teflon molds (4 × 4 × 4 cm), covered and sealed with a plastic sheet to prevent water evaporation, and stored at room temperature for 24 h. After the curing process, the cubic samples were de-molded and were stored at room temperature for 30 days before any testing. This process was repeated, and the mixtures were separately made with different calcined silt. The detailed mixture design is shown in Table 3. The amount of the materials was selected as such to satisfy the equation Na/Al = 1. This could reduce the leachate of the free sodium or potassium cations in the samples.

#### 2.2.2. Unconfined Compressive Strength

After 30 days of curing, the unconfined compressive strength of the cubic samples was evaluated through a hydraulic press (Galdabini, Italy). A constant loading speed was applied, and all the procedures were based on the EN 1015-11: 2019 standard. For each run, three replicates were tested. The average UCS was used for further calculations.

#### 2.2.3. Leaching Test

The horizontal dynamic surface leaching test (DSLT) was conducted in this study according to the CEN/TS 16637-2 standard proposed by the EC-CPR. The samples for the leaching tests (4 × 4 × 4 cm) were prepared with the same procedure described in Section 2.2 for geopolymers, with K1 and Na as liquid hardeners and calcination temperatures of 200, 295, 550, 750, and 850 °C. The samples were placed in glass tanks with sealed caps to prevent the liquid from evaporating. The space between the specimens and tank walls was more than 20 mm in all directions. Samples were placed on spacers at the bottom of the containers in order to have all of the sample sides in contact with the leachant. Deionized (DI) water was used as the leachant in this study. A sample surface to water volume of 80 L/m was chosen according to the CEN/TS 16637-2 standard. Leaching tests were conducted in 3 replicates prior to the statistical analysis of the results. The room and leachant temperature were controlled and maintained between 20–25 °C. The leachant in the containers was sampled and renewed at time intervals of 0.25, 1, 2.25, 4, 9, 16, 36, and 64 days with the duration of each step being 0.25, 0.75, 1.25, 1.75, 5, 7, 20, and 28 days, respectively. Control experiments were conducted with DI water in containers in the absence of geopolymer specimens.

Samples from leaching and control tests were collected to evaluate the concentrations of Al, As, B, Ba, Ca, Cd, Co, Cr, Cu, Fe, K, Mg, Mn, Mo, Na, Ni, Pb, Sb, Se, Si, Tl, V, and Zn. The pH and electric conductivity of the liquid samples were also measured for samples from all of the time intervals. Concentrations of the compounds were measured using a Perkin-Elmer Optima 5300 DV ICP-OES instrument. The following equation (Equation (1)) was used to calculate the normalized concentrations of the leached compounds:

(1) $$r_{i}=\frac{c_{i}\;V}{A}$$

where i is the sampling step, c_i_ is the concentration of the compound in the leachant (mg/L), V is the volume of the leachant (L), and A is the surface area of the specimens (m^2^).

The cumulative concentrations of the leached compounds (Equation (2)) were calculated as follows:

(2) $$C_{n}=\overset{n}{\underset{i=0}{\sum }}r_{i}$$

where C_n_ (mg/m^2^) is the cumulative concentration at the step n of leaching test, and i is the sampling step.

### 2.3. Geopolymer Binder Preparation and Testing Procedures (Phase II)

A second DOE (Table 4) was designed, aiming to compare the effect of different types of liquid activators and the amount of silt on the final geopolymer cement strength. For this purpose, two of the previous liquid hardeners (Na and K1) plus a third commercial potassium-based liquid (K2) were used (MR = 3.2). The amounts of silt selected were 150, 200, and 250 g. The amounts of MK for Na, K1, and K2 selected were 133, 101.9, and 54.66 g, respectively. For all mixtures, the liquid amount was selected as 192 g. The MK/liquid ratio was selected is such manner to fulfil Na(or K)/Al = 1.

A full factorial design having a total of 9 (3 × 3) randomized runs was produced. The model was produced using the standard least squares with emphasis on effect leverage. The samples were prepared as they were in the previous phase (Section 2.2.1), and the cubes were tested for compressive strength after 30 days of room temperature curing (EN 1015-11: 2019). Phase II of the study was independent of the first phase. However, the silt calcination conditions were taken from the first phase. Therefore, for all of the samples produced in the second phase, the silt was calcined at 750 °C for 2 h (further details are provided in the Discussion section). The detailed mixture design is presented in Table 5.

## 3. Results

### 3.1. Calcined Silt Mineralogical Characterization (Phase I)

A total of three samplings of the silt were performed to investigate the variations of the mineralogical composition in the sedimentation lakes. All of the samples were found to be composed of quartz, calcite, phyllosilicates with characteristic interplanar distances associable with chlorite, kaolinite/serpentine and illite/mica, and albite-like feldspar, as determined by means of XRD qualitative analysis. K-feldspar and traces of dolomite were also found, but they were not found in all of the samples.

The XRD semi-quantitative analysis revealed the presence of quartz and calcite as major constituents, with variability within about 30–40 wt% and 25–30 wt%, respectively. Significant amounts of illite/mica, chlorite, kaolinite/serpentine, and albite-like feldspar were also detected, with variability within about 12–20 wt%, 2–7 wt%, 5–11 wt%, and 4–12 wt%, respectively. Where present, K-feldspar were found up to about 9 wt%, whereas dolomite was found in small amounts up to about 2 wt%.

**Figure 5:**
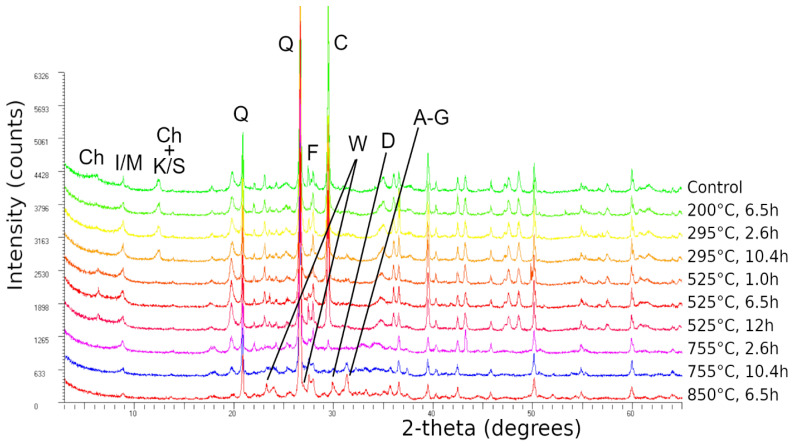
XRD profiles of the control and calcinated silt obtained with Cu Kα radiation. C = Calcite, Q = Quartz, W = Wollastonite, D = Diopside, A-G = Åkermanite–Gehlenite solid solution, I/M = Illite–Mica, Ch = Chlorite, K/S = Kaolinite/Serpentine, F = Feldspars.

After the mineralogical characterization of the raw material, thermal treatments (calcination) were conducted at different times and temperatures, as detailed in Table 2, to increase the reactivity of the silt. Figure 5 reports the X-ray diffractograms of the control and calcinated silt.

No variation in the mineralogical composition of the calcinated silt was revealed by XRD analysis at temperatures up to 295 °C, independent of the calcination time. The thermal treatments at 525 °C, independent of the treatment time, caused an increase in the X-ray intensity of the diffraction peak at about 14 Å, which was also associated with a slight shift to a higher angle, the complete collapse of the peak at about 7 Å, and no significant variation of the other peaks. The calcination at higher temperatures (i.e., 750 °C and 850 °C) caused the complete collapse of the diffraction peak at about 14 Å and a decrease in the intensity of the peak at about 10 Å (runs 10 h at 750 °C and 6.5 h at 850 °C). Still, the almost total collapse of the calcite peaks (which collapsed at 750 °C for 10 h and at 850 °C for 6.5 h) and the formation of new mineral phases were observed. In particular, the treatment at 750 °C for 2.6 h produced a wollastonite-type (CaSiO_3_) new phase, whereas the other two treatments also produced a diopside-like (CaMgSiO_2_O_6_) phase and a mineral in the Åkermanite (Ca_2_MgSi_2_O_7_)–gehlenite (Ca_2_Al(SiAl)O_7_) solid solution.

### 3.2. Binder Properties

#### 3.2.1. Unconfined Compressive Stress-DOE Analysis (Phase I)

The average UCS obtained for the samples prepared with Na or K1-based liquid hardeners are presented in Table 6. Overall, an increase in the calcination temperatures led to an increase in the final compressive strength of the geopolymer cement samples. This trend was true for the samples made with both Na- and K-based liquid hardeners. However, the effect of calcination time on the final UCS differed for low and high temperatures. In this regard, an increase in calcination time at lower temperatures (<525 °C) seemed to increase the final strength of the samples. However, at higher temperatures, an increase in the calcination time led to a decrease in the compressive strength. For instance, by increasing the time from 1 to 6.5 h, the compressive strength of the Na-based samples made with silt calcined at 525 °C increased. However, a further increase in calcination time led to a decrease in the final strength, with values from 49.40 to 43.57 MPa.

The obtained data were then inputted into JMP^®^ software, and a response surface method analysis was applied. The input variables were selected as calcination time and temperature, and the resulting UCS for both Na- and K-based geopolymers were selected as the two responses. The summary of the fit and parameter estimates for the Na-based geopolymers are tabulated in Table 7 and Table 8, respectively. The R-squared value of 93.08% indicated a high confidence fit of the model (Table 7). The data presented in Table 8 indicated a high impact of the temperature factor on UCS, with a t-ratio of 7.04 and high significance levels (<0.0021). However, time did not show a significant effect on the compressive strength.

**Figure 6:**
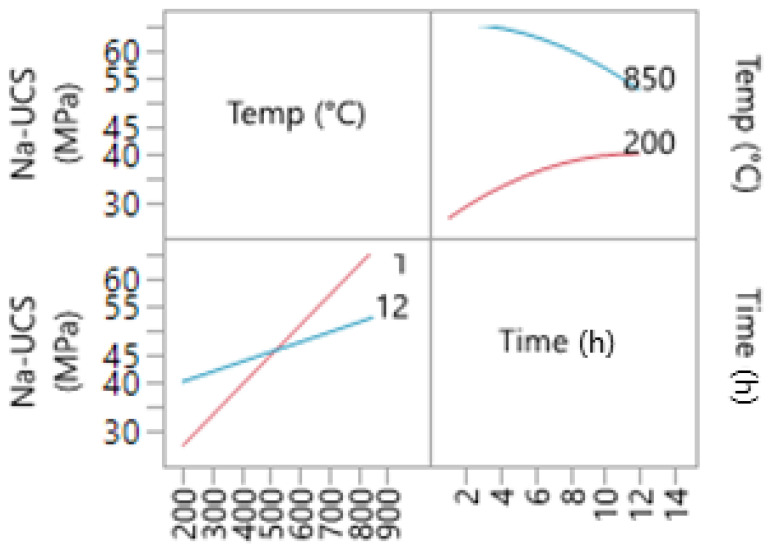
Interaction profile for time and temperature (Na-based liquid).

The interaction between calcination time and temperature for Na-based geopolymer cements is shown in Figure 6. The interaction also indicates that at higher temperatures, an increase in calcination duration could decrease the compressive strength of the samples. Moreover, the rate at which temperature impacts the UCS is higher when a lower calcination duration (1 h) is used compared to a higher calcination time.

**Figure 7:**
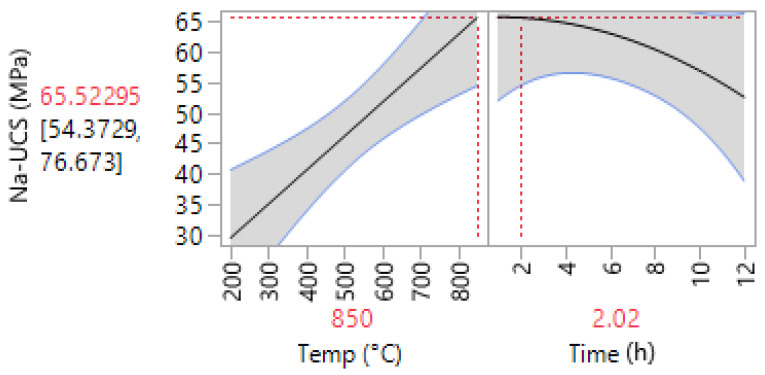
Prediction profiler for Na-based geopolymer cement.

**Figure 8:**
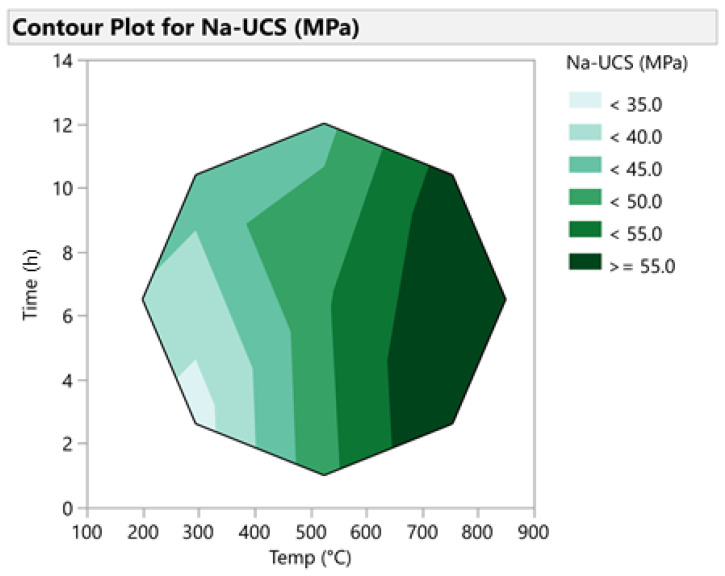
Contour plot for Na-based geopolymer cement.

The prediction profiler is shown in Figure 7. This tool allows for the optimization of the factors and outcomes based on different desirability factors. Thus, to achieve the highest compressive strength, the parameters were changed accordingly. Consequently, by minimizing the calcination duration to 2 h, the maximum compressive strength of 65.52 MPa was predicted. Figure 8 shows the contour plot for sodium-based geopolymer samples. As indicated, the highest compressive strength is achievable when calcination temperatures are higher than 650 °C.

**Figure 9:**
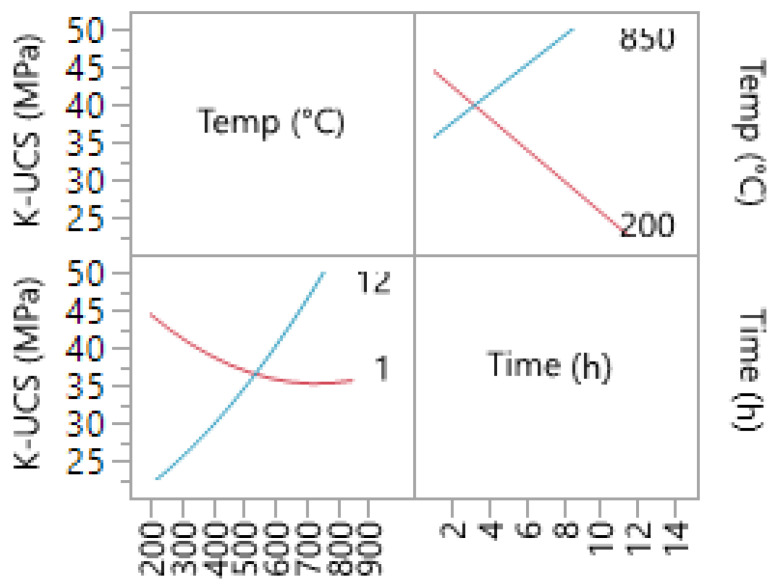
Interaction profile for time and temperature for K1-based samples.

The summaries of the fit and parameter estimates for the samples produced with potassium liquid hardeners are presented in Table 9 and Table 10, respectively. Similarly, the model had an R-squared value of 0.9116, indicating a high precision fit of the model. Again, the temperature is what has the highest impact on the final strength. However, for the potassium-based models, the interaction between time and temperature is significant (<0.05), with a positive interaction value of 4.03. Thus, as illustrated in Figure 9, an increase in both factors will result in the highest UCS possible.

**Figure 10:**
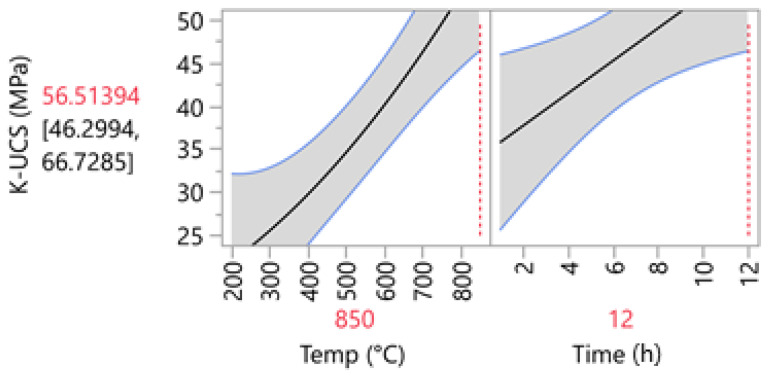
Prediction profiler for K1-based geopolymer cement.

**Figure 11:**
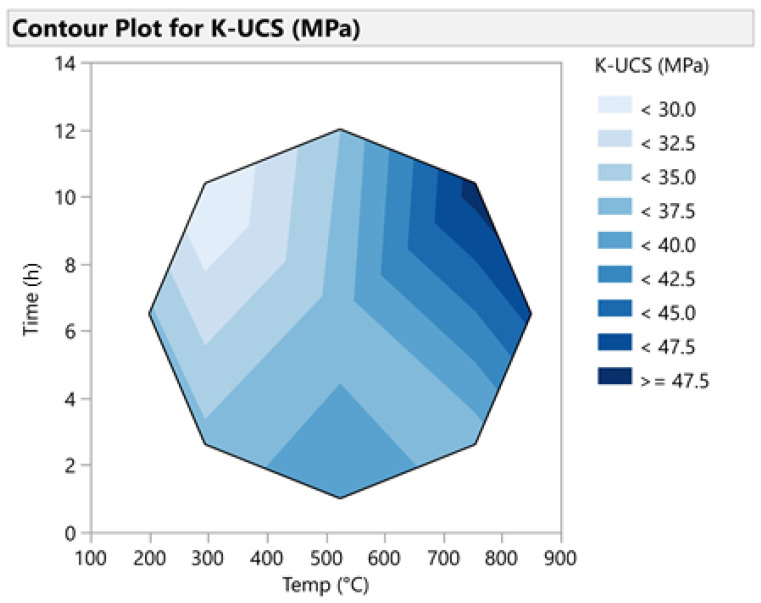
Contour Plot for K1-based geopolymer cement.

The prediction profiler for potassium-based geopolymer cements indicated that the highest UCS can only be achieved if the high temperature (850 °C) and long calcination duration of 12 h are used (Figure 10). Calcination completed in shorter durations will result in lower UCS values. This is also indicated by the contour plot (Figure 11), which indicates the requirement of both high temperature and duration for achieving high compressive strength values.

#### 3.2.2. Horizontal Dynamic Surface Leaching Test (Phase I)

**Figure 12:**
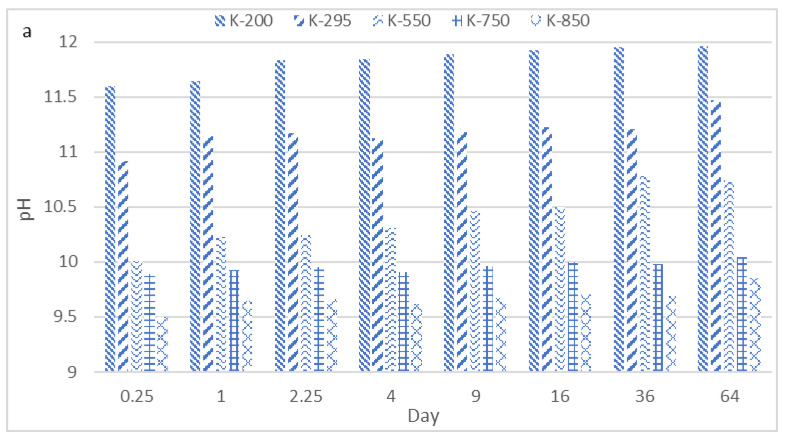

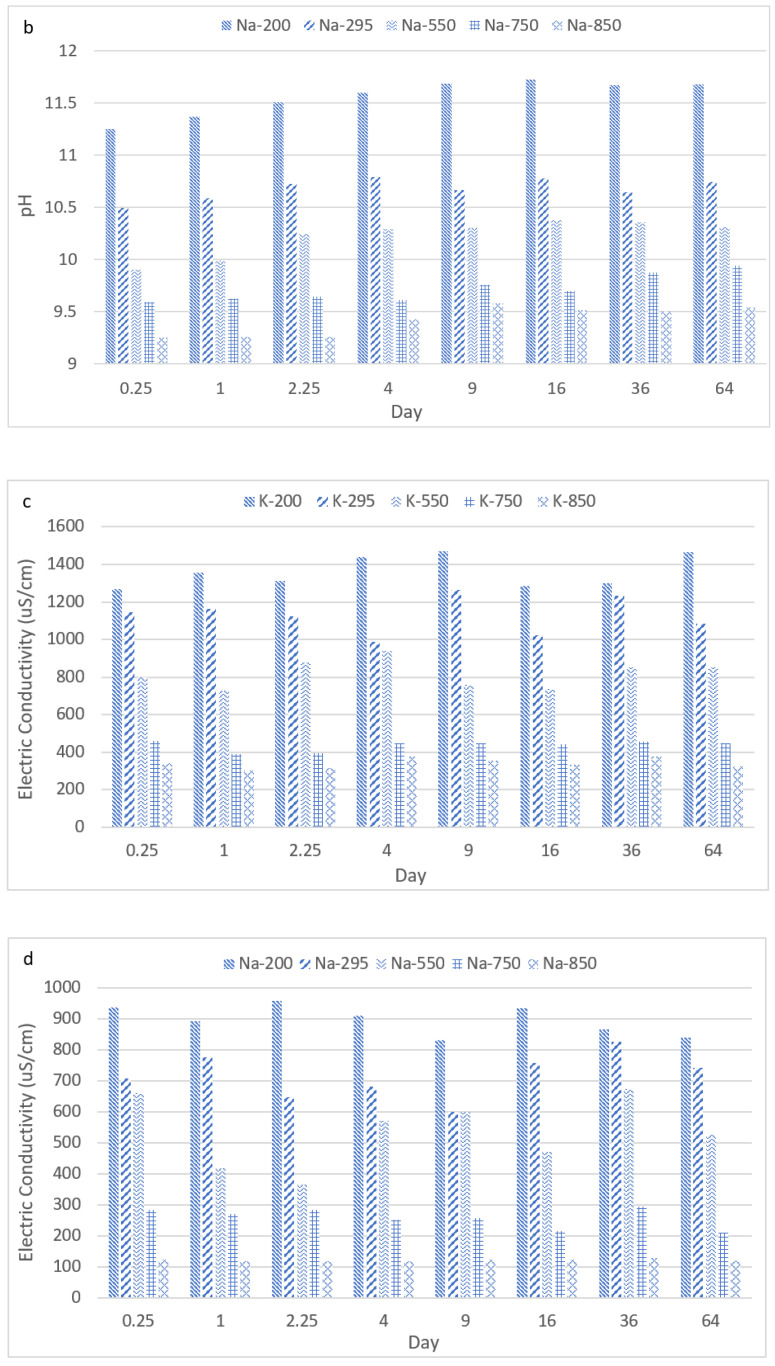
pH values (**a**,**b**) and electric conductivity (**c**,**d**) of leachants during the DSLT tests. K refers to the K1 liquid used in Phase I.

The changes in the pH values during the DSLT test for the K- and Na-based geopolymers are presented in Figure 12. The K-based geopolymers (K1) showed higher pH values than the Na-based specimens at all calcination temperatures. K-based geopolymers calcinated at 200, 295, 550, 750, and 850 °C showed a pH ranging between 11.6–11.9, 10.9–11.4, 10.0–11.7, 9.9–10.0, and 9.5–9.8, respectively. The pH values for the Na-based geopolymers ranged from 11.2–11.7, 10.5–10.7, 9.9–10.4, 9.6–9.9, and 9.2–9.5 at calcination temperatures of 200, 295, 550, 750, and 850 °C, respectively.

The evolution of the electric conductivity of K- and Na-based geopolymers was evaluated using the DSLT test and was reported for different time intervals from 0.25 to 64 days (Figure 12). According to the results, the mean value of electric conductivity for leachant in contact with K-based geopolymers was between 1269–1466, 988–1263, 736–940, 392–461, and 300–376 uS/cm depending on the DSLT time interval. The Na-based specimens showed an electric conductivity of 839–957, 601–825, 365–673, 211–295, and 119–129 uS/cm for calcination temperatures of 200, 295, 550, 750, and 850 °C, respectively.

The cumulative concentration of released heavy metals and trace elements per unit of K- and Na-based geopolymer specimen surface are presented in Table 11. According to the results, no concentrations of Se, Tl, and V were observed in K- or Na-based geopolymer leachants during the DSLT test. Moreover, for the K-based geopolymers calcinated at 850 °C, the concentrations of Cd, Ni, Pb, and Sb were below the instrument detection limit for all of the DSLT time intervals. No concentrations of Cd, Co, Mn, Pb, or Sb were leached to the DI water from the Na-based geopolymers calcinated at 750 and 850 °C during the DSLT test. Additionally, the leachants from the Na-based geopolymers calcinated at 850 °C contained concentrations of Cu and Ni that were below the detection limit. The cumulative release of Al, Ca, K, Na, and Si in the leachants during the DSLT tests ranged between 53–1257, 12–147, 1300–36,858, 9–175, and 1648–19,586 mg/m^2^ for the K-based geopolymers, respectively. Cumulative concentrations of Al, Ca, K, Na, and Si of 81–1068, 7–115, 7–95, 2390–29,774, and 1148–16,648 mg/m^2^ o were detected in the leachants from the Na-based geopolymers in the DSLT test, respectively.

### 3.3. Effect of Silt and Activator Type on UCS (Phase II)

**Figure 13:**
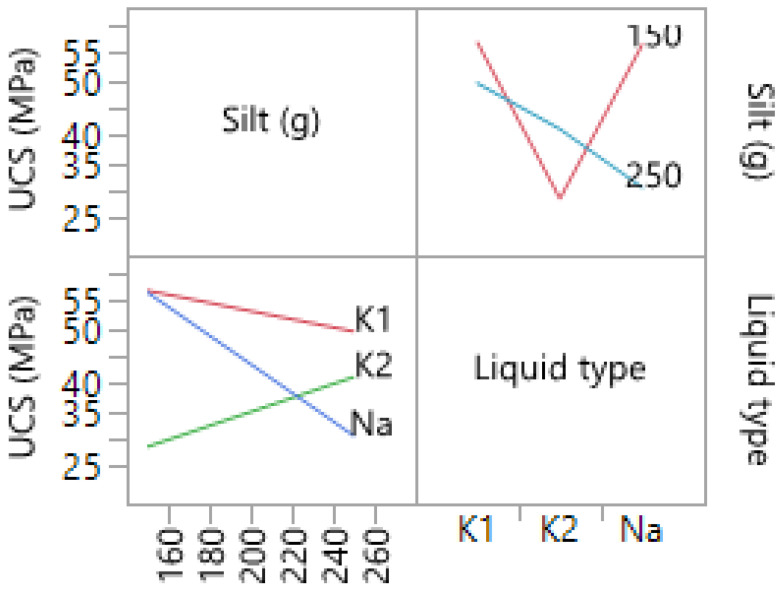
Interaction plots of samples produced with different silt amounts and liquid types.

The compressive strength correlated to the percentage of silt and liquid hardener type is shown in Table 12. The liquid types of Na, K1, and K2 refer to the sodium-based and two potassium-based liquid hardeners. The highest UCS of 57.46 MPa was reported for the samples made with K1 liquid, whereas the lowest value was reported for the K2 sample with 29.05 MPa. The silt amounts used for K1 and K2 were reported as 59.5 and 72.7% (total solid weight), respectively. The data were further analyzed in JMP^®^ software, providing more information regarding the effect of the silt and liquid type on the final mechanical strength of the samples. The interaction plots (Figure 13), further show the correlation between the studied factors and the output response. With an increase in the silt amount, the final compressive strength of the samples made with Na and K1 liquids decreased. However, for the samples made with K1 liquids, the compressive strength decrease is not as high as the ones for the Na liquids, where a loss of about 20 MPa was observed when the amount of silt was increased from 53 to 65.3% (total solid weight). The only samples that showed an increase in strength with an increase in silt content were the samples made with K2 liquid hardener. The produced model had an R^2^ = 0.994, which was shown to be significant (*p* < 0.0013).

## 4. Discussion

The results from the mineralogical analysis of the raw silt evidenced small variability in the silt composition, mainly in terms of the amount of the mineral phases rather than the type of phases. Only dolomite and K-feldspar were occasionally observed: the first was detected in just a few weighted percentages, and the second was detected up to about 9%; however, it was not affected by the calcination treatments and thus, in principle, did not participate in the change of reactivity of the silt. The variability is an important parameter for the mass production of samples. This could alter the final strength of the produced samples due to variation in the phase of the waste silt. However, the results did not show, nor did they affect the current test results since no variation in the phase was observed.

As expected, the XRD profile was not affected by the treatments for temperatures up to 295 °C. The lowest values for compressive strength, regardless of liquid type, were achieved at calcination temperatures below 295 °C. Based on the leaching results, low calcination temperatures lead to the undesirable leaching of alkali cations (Na^+^ or K^+^) and for all cases, sodium-based samples showed lower leaching values than potassium-based mixtures. The low UCS values and high leaching could be associated with the inert behavior of the silt calcined at low temperatures.

The variation of the XRD profile for calcination at 525 °C was consistent with the dehydroxylation of the interlayer hydroxide of the chlorite and the dehydroxylation of kaolinite/serpentine, regardless of the calcination time. This increased the reactivity of the silt and consequently increased the compressive strength for both the sodium and potassium-based mixtures (Table 6).

The treatments at about 750 °C for 2.6 h also caused the dehydroxylation of the talc-like layer of chlorite and its complete amorphization as revealed by XRD, the almost total loss of calcite, and the formation of a wollastonite-type (CaSiO_3_) new phase. All of these types of transformations are well reported and described by Földvari (2011) [31]. The last two treatments, 750 °C for 10 h and 850 °C for 6.5 h, also determined the complete loss of calcite, a progressive dehydroxylation of illite/mica, and the formation of a diopside-like (CaMgSiO_2_O_6_) phase and a mineral in the Åkermanite (Ca_2_MgSi_2_O_7_)–gehlenite (Ca_2_Al(SiAl)O_7_) solid solution in percentages up to about 10% [31].

The effectiveness of promoting aluminosilicate dissolution is higher in Na-based alkali solutions compared to K-based ones. Moreover, the viscosity of Na is much higher than K, making it harder to mix sodium-based geopolymer mixtures. However, based on the literature, K-based geopolymers should have higher compressive strength than Na-based samples, indicating that the rate of dissolution does not control the geopolymerization [15].

The results presented in Table 6 indicate that for all of the calcination conditions, the UCS obtained for Na-based mixtures are higher than the ones of the K-based samples. This could be related to the amount of silt used in the mixture. Only 100 g of silt (49.5% total solid weight) was added to the K1 samples during phase I, producing a maximum strength of 48.82 MPa using silt calcined at 750 °C for about 10 h. The silt acts as a partially reactive filler in the geopolymer mixture. For samples produced with K1, the filler amount was not sufficient, leading to the samples prone to cracking due to their brittleness. Generally, samples produced with metakaolin only will be very brittle unless a proper amount of filler is added to the mixture [32]. This could also be backed up with the results obtained during phase II of the study, where the effect of silt amount was investigated on the final strength of the samples. Table 12 indicates that the highest amount of UCS was obtained for the K1 samples produced with 150 g (59.5% total solid weight) of silt. Thus, increasing the amount of silt by 10%, an increase of approximately 10 MPa was observed for the K1 samples. However, by further increasing the amount of silt to 71%, the final strength slightly decreased to 50.10 MPa. This could be related to the fact that for a higher silt amount, water (less than 2% of total solid part) was added to the mixture, decreasing the mixture viscosity. Water could decrease the maximum strength of the samples by decreasing the molarity of the alkali solutions [15]. Moreover, an excess amount of filler (silt) could remain unreacted in a mixture and could behave as an inert material. This could produce micropores inside the samples, which can decrease the final strength of the mixtures [33]. The results for phase II were in line with the literature since the UCS for K1 was higher than the ones for Na, regardless of silt amount. Both the Na and K1 samples experienced an increase in the strength when the silt amount was increased from 150 to 250 g, whereas the strength of the K2 samples increased with an increase in the silt content. K2 had the lowest amount of viscosity amongst all of the liquid hardeners, allowing more silt to be used within the mixture. Thus, by adding a higher amount of silt (>250 g), the final compressive strength could exceed 41.68 MPa, as reported in Table 12.

The results of the DSLT tests revealed that the pH of the leachants from the K-based and Na-based geopolymers increased with the reduction of the geopolymer calcination temperature at each test time interval (Figure 12). The lowest and highest observed pH were associated with Na-based geopolymers calcinated at 850 °C (9.2) and 200 °C (11.7), respectively. The same pattern was observed with the K-based geopolymers. The increasing of the pH values was due to the leaching of the alkaline elements into the leachant [34,35]. With increasing calcination temperatures for both the K- and Na-based geopolymers, the number of alkaline elements participating in the geopolymerization process and creating bonds in the potassium and sodium alumina–silicate gels increased [36]. This phenomenon resulted in lower rates of alkaline element release into the leachant and consequently lower pH values. This observation was in line with the mechanical results, where the geopolymers calcinated at higher temperatures showed higher geopolymerization rates and UCS values (Table 6).

The electric conductivity values of the leachants from the DSLT tests for the K- and Na-based geopolymers increased with decreasing calcination temperatures (Figure 12). This result was due to the higher release rate of Na and K ions into the leachant, which resulted in higher values of electric conductivity for the geopolymers calcinated at lower temperatures [37]. The maximum and minimum electric conductivity for the K-based geopolymers were 1468 (200 °C) and 300 (850 °C) uS/cm, respectively.

The results of the DSLT tests revealed that the cumulative concentrations of released heavy metals and trace elements into the leachant solution during the DSLT tests were influenced by the geopolymer calcination temperature and pH of the leachant. According to Table 11, the concentration of all of the released elements reduced by increasing the calcination temperature. The cumulative released concentrations of the elements, including Cd, Ni, Pb, and Sb, from the K-based geopolymer surface and of Cd, Co, Mg, Ni, Pb and Sb for the Na-based geopolymers, reduced constantly with the increase in the calcination temperature, to a point where no ions of concern were detected (750 and 850 °C). The same pattern was observed for other elements, with a reduction up to 96% for the cumulative released concentrations from the surface unit of the geopolymers. This observation may be explained by two factors. First, as described earlier, a more complete degree of geopolymerization occurred at higher calcination temperatures for both the Na- and K-based geopolymers. As a result, trace elements had stronger bonds with the structure of the geopolymer, which led to lower rates of release into the leachant [38]. Second, as described earlier, the pH of the leachant in contact with the geopolymers calcinated at lower temperatures were higher than that of the geopolymers calcinated at lower temperatures. The high pH may have increased the mobility of the metals [12,13,39,40], and the trace elements, which resulted in higher rates of release into the leachant during the DSLT tests.

Overall, the DSLT leaching test results revealed that the cumulative release of heavy metals and trace elements, including As, B, Ba, Ca, Cd, Co, Cr, Cu, Fe, K, Mg, Mn, Mo, Na, Ni, Pb, Sb, Se, Si, Tl, V, and Zn, from the Na-based and K-based geopolymers under investigation in the present study were in the same range as other reported studies on geopolymer mortars and cement-based materials [41]. Moreover, the cumulative release of elements per unit surface of the geopolymers were lower than thresholds of the EU Water Framework Directive (The Water Framework Directive 2000/60/EC).

From a mechanical point of view, every sample, regardless of calcination condition and liquid type, showed compressive strength above 30 MPa. This could be comparable to the strengths obtained for ordinary concrete mixtures used in the construction sector. However, calcination temperatures below 750 °C showed excess leaching of alkali cations. Moreover, new mineral phases were only observable for calcination temperatures of 750 and 850 °C. Thus, the optimum calcination temperature was selected as 750 °C for all liquid types satisfying mineralogical, mechanical, and environmental criteria. The optimum amount of silt was selected as 53 and 59.5% (total solid part) for the Na and K1 samples, respectively. However, more than 81.6% silt could be added to the samples produced with K2 liquid, which had the lowest viscosity. Recycling as much silt as possible could be environmentally friendly and could provide a circular economy for S.A.P.A.B.A. s.r.l.

To sum up, calcination of silt appears to be a promising option for increasing the final strength of the sample, allowing the reuse of the aggregate production by-product, silt.

## 5. Conclusions

The current study investigated the feasibility of using thermally treated waste silt obtained from S.A.P.A.B.A. s.r.l. as filler in geopolymer cement production. The data obtained from XRD indicated a presence of 43.5% and 12.5% of SiO_2_ and Al_2_O_3_, respectively. However, the mineralogical data indicated a high crystallinity rate of the silt, which was composed of minerals including quartz, calcite, phyllosilicates with characteristic interplanar distances associable with chlorite, kaolinite/serpentine, illite/mica, feldspar such as albite and K-feldspar, and traces of dolomite. Therefore, various thermal treatment conditions were applied, and their effect on the final strength of the samples were studied. Leaching tests were conducted to further study the behavior of the calcined silt.

Based on the presented results, the following conclusions can be drawn:

- Overall, higher calcination temperatures led to higher compressive strength values.
- Low calcination temperatures (<250 °C) did not change the mineral compositions of the calcined silt.
- Phase change occurred at higher calcination temperatures, leading to the dehydroxylation of the chlorite at 550 °C and the complete loss of calcite (T > 750 °C).
- DSLT leaching test results showed that increasing the calcination temperature of the K- and Na-based geopolymers resulted in the lower cumulative leaching of heavy metals and trace elements.
- The concentrations of the released elements from the geopolymer specimens were in the same range as cement-based materials and were lower than those of the EU Water Framework Directive thresholds.
- An excess amount of filler could decrease the compressive strength of the final mixture.
- The optimum amount of silt was selected as 53 and 59.5% of the total solid part for the Na and K1 liquids, respectively.
- The K2 liquid had the lowest viscosity compared to the other liquids, allowing the addition of more than 81.6% of silt into geopolymer cement samples.
- The optimum calcination temperature was selected as 750 °C for all liquid types, satisfying mechanical, mineralogical, and environmental criteria.

## Figures and Tables

**Table 1:** Chemical composition of uncalcined silt.

Parameter	Value (%)
SiO_2_	43.5
TiO_2_	0.6
Al_2_O_3_	12.5
Fe_2_O_3_	6.1
MnO	0.2
CaO	15.8
Na_2_O	1.0
K_2_O	1.9
P_2_O_5_	0.1
MgO	3.0

**Table 2:** DOE for silt calcination under different times and temperatures.

Run Order	Temp (°C)	Time (h)
1	200.0	6.5
2	295.2	10.4
3	754.8	10.4
4	295.2	2.6
5	850.0	6.5
6	525.0	6.5
7	525.0	12.0
8	754.8	2.6
9	525.0	1.0

**Table 3:** Detailed mixture design for geopolymer cement production.

Liquid Hardener Type-Amount (g)	MK/Liquid Hardener	MK (%) *	Silt (%) *
Na-192.0	0.69	65.5	34.5
K1-192.0	0.53	50.5	49.5

* Total solid weight (MK + silt).

**Table 4:** The effect of silt amount and activator type on UCS (DOE).

Run Order	Activator	Silt (g)	Silt (%) *
1	Na	150	53
2	K1	250	78
3	Na	250	65.3
4	K2	200	71
5	K2	250	81.6
6	K1	150	59.5
7	K2	150	65.3
8	K1	200	60.2
9	Na	200	60.1

* Total solid weight (MK + silt).

**Table 5:** Mix design proportions for liquid hardener and MK.

Liquid Hardener Type-Amount (g)	MK/Liquid Hardener
Na-192.0	0.69
K1-based-192.0	0.53
K2-based-192.0	0.29

**Table 6:** Compressive strength obtained for different calcination conditions (DOE).

Run Order	Temperature (°C)	Time (h)	Na-UCS (MPa)	K1-UCS (MPa)
1	200.0	6.5	39.43	35.49
2	295.2	10.4	42.15	27.00
3	754.8	10.4	57.58	48.82
4	295.2	2.6	32.50	35.85
5	850.0	6.5	59.52	45.85
6	525.0	6.5	49.40	36.24
7	525.0	12.0	43.57	34.98
8	754.8	2.6	60.81	35.96
9	525.0	1.0	48.52	39.59

**Table 7:** Summary of fit (Na-based liquid).

Statistical Term	Value
RSquare	0.930802
RSquare Adj	0.861603
Root Mean Square Error	3.621467
Mean of Response	48.16444
Observations (or Sum Wgts)	9

**Table 8:** Parameter estimates (Na-based liquid).

Term	Estimate	Std Error	t Ratio	Prob > |t|
Intercept	49.636366	1.891252	26.25	<0.0001
Temp (°C) (200,850)	12.754706	1.810732	7.04	0.0021
Time (h) (1.12)	−0.10259	1.81073	−0.06	0.9575
Temp (°C) × Time (h)	−6.439971	3.62145	−1.78	0.1500
Time (h) × Time (h)	−3.311813	3.275738	−1.01	0.3692

**Table 9:** Summary of fit for K1-based samples.

Statistical Term	Value
RSquare	0.911692
RSquare Adj	0.823385
Root Mean Square Error	2.694961
Mean of Response	37.75333
Observations (or Sum Wgts)	9

**Table 10:** Parameter estimates for K1-based samples.

Term	Estimate	Std Error	t Ratio	Prob > |t|
Intercept	36.247273	1.407398	25.75	<0.0001
Temp (°C) (200,850)	6.4667096	1.34748	4.80	0.0087
Time (h) (1.12)	−0.443622	1.347478	−0.33	0.7585
Temp (°C) × Temp (°C)	3.3886312	2.437683	1.39	0.2369
Temp (°C) × Time (h)	10.854951	2.694949	4.03	0.0158

**Table 11:** The cumulative concentrations of the released heavy metals and trace elements from the surfaces of the geopolymer specimens (mg/m^2^).

Element	K-Based Geopolymer Specimens					Na-Based Geopolymer Specimens				
	K-200 *	K-295	K-550	K-750	K-850	Na-200	Na-295	Na-550	Na-750	Na-850
Al	1257.55	578.473	231.38	127.26	53.45	1068.91	587.90	317.46	139.68	81.017
As	5.62	2.75	1.15	0.56	0.28	4.66	2.19	0.92	0.44	0.26
B	73.45	38.19	19.09	7.63	3.81	53.61	23.05	9.91	5.65	3.05
Ba	13.84	5.53	2.21	0.93	0.39	11.21	4.59	2.52	1.39	0.62
Ca	147.56	78.20	46.92	23.46	12.90	115.09	63.30	37.34	16.43	7.06
Cd	0.66	0.28	0.13	0.06	ND	0.56	0.28	0.16	ND **	ND
Co	0.81	0.39	0.16	0.09	0.04	0.55	0.27	0.14	ND	ND
Cr	3.93	1.65	0.94	0.45	0.25	3.06	1.44	0.72	0.38	0.15
Cu	0.92	0.37	0.21	0.12	0.07	0.77	0.43	0.24	0.12	ND
Fe	103.57	56.96	27.34	12.30	4.92	83.89	50.33	24.66	10.35	6.01
K	36,858.1	15,480.41	6501.77	2600.71	1300.35	95.14	51.37	30.31	13.03	7.69
Mg	7.69	4.22	1.86	0.93	0.38	5.07	2.89	1.47	0.73	0.42
Mn	1.12	0.56	0.25	0.11	0.06	0.84	0.45	0.22	ND	ND
Mo	17.45	7.67	3.07	1.56	0.73	11.69	6.19	3.47	1.49	0.68
Na	175.34	82.41	43.67	19.21	9.03	29,774.44	15,184.96	8351.73	4509.93	2390.26
Ni	0.77	0.43	0.26	0.10	ND	0.56	0.31	0.18	0.08	ND
Pb	0.08	0.04	0.01	ND	ND	0.06	0.03	0.01	ND	ND
Sb	0.11	0.06	0.02	0.015	ND	0.09	0.05	0.02	ND	ND
Se	ND	ND	ND	ND	ND	ND	ND	ND	ND	ND
Si	19,586.1	9988.93	5493.91	3296.34	1648.17	16,648.21	8324.10	3662.60	2051.06	1148.59
Tl	ND	ND	ND	ND	ND	ND	ND	ND	ND	ND
V	ND	ND	ND	ND	ND	ND	ND	ND	ND	ND
Zn	67.19	33.59	14.10	5.92	2.42	49.04	21.09	11.60	6.72	2.96

* K-200: K-based geopolymer calcinated at 200 °C; ** ND: Not detected. K-based geopolymers refers to samples made with K1 liquid in Phase I.

**Table 12:** UCS for samples produced with different amount of silt and liquid type.

Liquid Type	Silt (g)	Silt (%) *	UCS (MPa)
Na	150	53	56.00
K1	250	78	50.10
Na	250	65.3	30.07
K2	200	71	34.07
K2	250	81.6	41.68
K1	150	59.5	57.46
K2	150	65.3	29.05
K1	200	60.2	52.08
Na	200	60.1	44.78

* Total solid weight (MK + silt).

## Data Availability

All data have been provided within the article.
